# Behaviour change solutions driven by cognitive insights for improving TB health care seeking among vulnerable population: an exploratory multi-state qualitative study in India

**DOI:** 10.1186/s12889-026-26542-x

**Published:** 2026-02-12

**Authors:** Atreyee Sinha, L. Ponnuchamy, Ambuja Kowlgi, Arin Kar, Karikalan Nagrajan, Rajaram Subramanian Potty, Shramana Majumder, Rehana Begum, Joseph Francis Munjattu, Gobinda Majhi, Arathi Rao, Reuben Swamickan, Amar Shah, Karthikeyan Kumarasamy

**Affiliations:** 1 Karnataka Health Promotion Trust (KHPT), Bengaluru, Karnataka 560044 India; 2 National Institute of Mental Health and Neurosciences (NIMHANS), Bengaluru, Karnataka 560029 India; 3 ICMR-National Institute for Research in Tuberculosis (NIRT), Chennai, Tamil Nadu 600031 India; 4 Department of Global Public Health Policy and Governance, Prasanna School of Public Health, Manipal Academy of Higher Education (MAHE-PSPH), Manipal, Karnataka 576104 India; 5 Health Office, USAID, New Delhi, India

**Keywords:** Tuberculosis, Behaviour change solutions, Cognitive, Healthcare seeking, Vulnerable populations, India

## Abstract

**Background:**

Tuberculosis disproportionately affects vulnerable groups due to low health awareness and suboptimal care-seeking behaviour. Individualised person-centric approaches are scarce in India’s national TB program. This study assessed the usefulness of behaviour change solutions (BCS) in improving TB case identification and treatment adherence among mining/industrial workers, migrants, tea-garden workers, tribals, and urban vulnerable.

**Methods:**

In this qualitative study we conducted 147 in-depth interviews (IDI) with adult (78 men, 69 women) BCS beneficiaries, facilitators (auto drivers, community leaders) and healthcare providers between May–August, 2023 in Karnataka, Telangana, Assam, and Bihar. We explored the usefulness of four BCSs — health auto (addressing decision fatigue and healthcare costs), jaanch coupon (addressing the loss aversion bias), TB starter-kit (reducing ambiguity aversion), and TB mukt certificate (reinforcing positive perceptions), through user experience. Interviews were conducted in local language (Kannada, Telugu, Hindi and Assamese), audio recorded, transcribed, and analysed using a deductive approach. A thematic analysis was done, focusing on participants’ knowledge of the solutions, their experiences using them, perceived benefits and challenges, and recommendations for improvement. Thematic saturation was reached through a process of collaborative analysis, where the research team iteratively reviewed and discussed emerging patterns. The study received ethics approval from the National Institute of Mental Health and Neurosciences (NIMHANS), Bangalore.

**Results:**

The initial codes were merged into major themes based on their similarity. Findings indicated that health auto and jaanch coupon enhanced TB related awareness at the community level, reduced diagnostic delay, and facilitated increased case finding. On the other hand, use of starter-kit and TB mukt certificate led to improved treatment adherence. BCSs also supported timely decision-making, promoted treatment adherence, and empowered persons with TB by fostering a sense of entitlement. Moreover, BCSs attempted to address multifaceted vulnerabilities: while case finding solutions were able to facilitate accessibility and affordability by addressing physical barriers, the case holding solutions reduced stigma and improved community acceptance. However, the data also indicated that the solutions had challenges related to acceptability, and scalability.

**Conclusions:**

Findings from this study underscored that BCS catalysed TB healthcare seeking by addressing cognitive barriers. It suggests that designing targeted cognitive interventions could potentially be a promising approach to alter cognitive biases, inculcate behavioural changes, and improve TB care-seeking and treatment outcomes.

**Supplementary Information:**

The online version contains supplementary material available at 10.1186/s12889-026-26542-x.

## Background

Tuberculosis (TB) remains a significant global health challenge, particularly in India which accounts for nearly one-quarter of the global burden [1]. The burden of TB in India is skewed and falls disproportionately on marginalized populations. One key challenge in mitigating the TB related morbidity and mortality, especially among marginalized communities is structural barriers including poverty, accessibility, and stigma in combination with poor living conditions, malnutrition, social deprivation, and social exclusion that lead to delayed diagnosis and poor adherence [2, 3]. India’s National Tuberculosis Elimination Program (NTEP) has made substantial progress in combating TB. However, challenges remain in achieving optimal control. Low case detection rates, delayed treatment initiation, lack of adherence to treatment regimens, higher rates of stigma and poor health seeking behaviour overall, remain critical barriers to effective TB management amongst these groups. These challenges are often intertwined with complex social, economic, and cultural factors that influence knowledge, attitude, health-seeking behaviours and treatment outcomes [4]. Each group has its own limitations when it comes to accessing care and completing treatment, influenced by their living conditions and social contexts. These differences underscore the need for personalized approaches for care and support. Therefore, targeting interventions to the most vulnerable groups may be necessary to accelerate progress toward eliminating this scourge [5]. Studies suggest that the delay in testing and discontinuation of treatment are not only due to structural barriers, but also a result of complex psychosocial processes that shape health-related decisions [6, 7]. Principles of cognitive and behavioural economics explain how loss aversion can make individuals reluctant to seek care if perceived costs outweigh uncertain benefits. Similarly, decision fatigue and ambiguity aversion undermine rational decision-making [7]. While principles of behavioural economics and cognitive psychology have been widely used in HIV/AIDS prevention and vaccination uptake [8, 9], its systematic application remains limited globally, particularly for TB care in India. In this context, behaviour change communication (BCC) strategies emerged as a potential tool to address various challenges by influencing knowledge, attitudes, and behaviours related to TB prevention, diagnosis, and treatment [10]. Effective communication strategies are crucial to render person-centred public health responses and promote positive health [11, 12]. Behaviour change and community centric interventions play significant role in communicable disease prevention such as HIV/AIDS, STIs etc [13, 14].

While India’s national strategic plan (NSP, 2017-25) for TB promotes community engagement through advocacy, awareness, various monetary incentives, and speaks of positive impact of effective communication in changing behaviours and attitudes, but an individualized approach addressing behaviour of vulnerable population to improve TB healthcare seeking and adherence may be required [15]. Hence a multi-state exploratory study was conducted with the question whether behavioural solutions based on theories of cognitive and behavioural sciences can possibly influence the health seeking behaviour among specific vulnerable communities. This paper aims to describe and assess the usefulness of the BCS solutions in facilitating TB notification, treatment adherence, and treatment completion among vulnerable populations at risk of TB.

## Methods

**Fig. 1:**
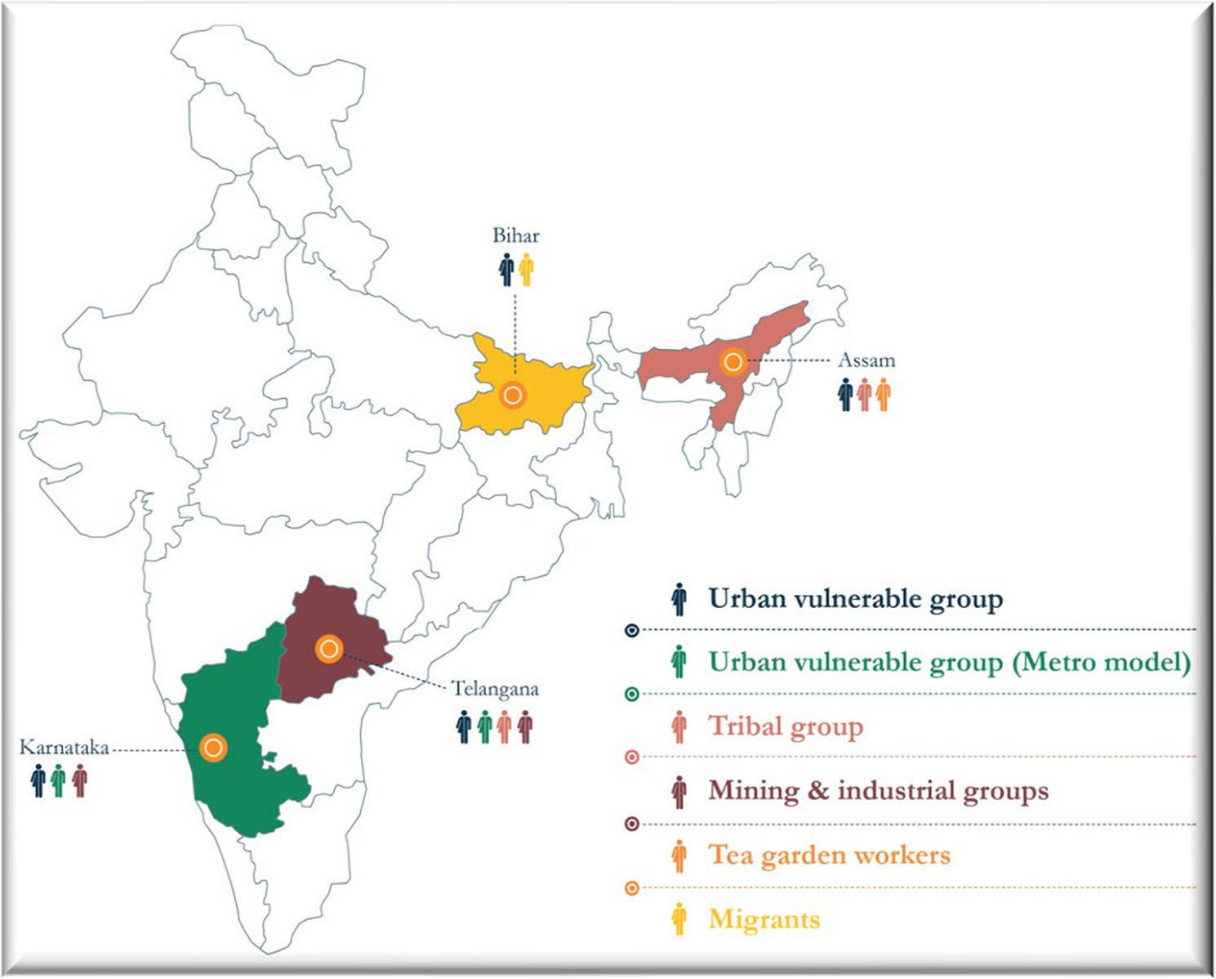
Geographical coverage of Breaking the Barriers Project

This paper used data from a qualitative research conducted as part of the USAID-supported Breaking the Barriers (BTB) project- a community engagement initiative to accelerate TB elimination in India, which aimed to increase TB case notification, improve treatment adherence and outcomes for drug-sensitive/drug-resistant TB among vulnerable groups (tribals, migrants, mining/industrial workers, tea garden workers, and urban vulnerable populations) in four Indian states: Karnataka, Telangana, Assam, and Bihar (Fig. 1).

As part of the BTB project, innovative tools also termed as behaviour change solutions (BCS) were developed through a co-creation method to facilitate TB health seeking and treatment adherence among vulnerable groups. The BCSs were implemented during October 2021-December 2023, in the four intervention states [16]. The four BCS concepts included in this study are described below:

### Operational definitions

#### Health auto

A free on-demand transport service linking symptomatic individuals and persons with TB (PwTB) to testing and treatment facilities. By reducing decision fatigue and hidden costs, it aimed to make TB care accessible and affordable. The service also leveraged action bias and inaction guilt, prompting timely health visits and sustaining motivation to engage with treatment.

#### Jaanch coupon

A referral slip distributed by health workers and community structures, offering a free TB test within seven days. By leveraging loss aversion bias, it created urgency and scarcity to motivate timely testing. The coupon also applied gain framing and a sense of ownership over government services, encouraging individuals to seek TB care despite stigma or hesitation.

#### TB starter kit

The kit included basic TB information and a treatment calendar, distributed and explained by health staff or community coordinators. Step-by-step instructions reduced ambiguity aversion, phase-wise milestones applied the goal gradient hypothesis, and daily reminders with helpline numbers leveraged the sunk cost fallacy/IKEA effect. By simplifying tasks and structuring progress, the kit reduced cognitive load and countered the ostrich effect, fostering treatment control.

#### TB mukt certificate

A certificate issued by health authorities to confirm successful treatment completion, usually distributed by medical officers during monthly care & support group meetings. It reinforced positive perceptions by addressing ambiguity aversion, offering assurance and closure, and leveraged the peak-end rule by marking the treatment journey with a memorable positive conclusion that strengthened motivation for adherence.

While, formal and informal community-based structures such as - women’s self-help groups (SHG), labour unions, faith-based organizations (FBO), community-based organizations (CBO), auto unions etc., were leveraged to roll out health auto and jaanch coupon, the other two solutions were implemented through care & support groups (CSGs) - a platform for inclusive gathering of PwTB and caregivers to improve patient-provider communication, and provide support to PwTBs at primary health institute (PHI) level.

### Setting and participants

A qualitative research was conducted between May-August 2023 in the tuberculosis units (TU) of four intervention states to understand the usefulness of BCSs in the community. A total of 147 in-depth interviews (IDIs) were conducted with adults (18+) who used the BCSs, facilitators such as auto drivers, and community structure leaders, and healthcare providers [different cadre of NTEP staff and Accredited Social Health Activists (ASHA)]. Sampling was based on achieving saturation across relevant categories rather than on state-wise representation, ensuring comprehensive coverage of experiences without aiming for geographic proportionality. Data was collected from 74 PwTBs, 10 caregivers, 37 NTEP staff (medical officers, senior treatment and lab supervisors, TB health visitors) and ASHA workers, 18 community structure (CS) leaders and eight auto drivers (Tables 1 and 2).

**Table 1:** In-depth interviews (IDI) conducted across the States by behaviour change solutions

IDI sample – BCS & state-wise					
	KA	TS	BH	AS	Total
Health Auto	10	15	5	10	40
Jaanch Coupon	12	12	4	12	40
TB Mukt Certificate	9	6	3	9	27
Starter Kit	12	12	4	12	40
Total	43	45	16	43	**147**

*KA* Karnataka, *TS* Telangana, *BH* Bihar, *AS* Assam

**Table 2:** Sample characteristics of the in-depth interviews (IDIs)

		Percentage distribution (%)
Gender	Male	52.9
	Female	47.1
Age groups (in years)	18–25	10.3
	25–35	29.7
	35–45	26.5
	45–55	17.4
	55+	12.9
	Not disclosed	3.2
Education	Illiterate	9.7
	Below primary/Primary	9.7
	Secondary	20.6
	Higher secondary & above	48.4
	Not disclosed	11.6
Religion	Hindu	54.2
	Muslim	10.3
	Others	0.6
	Not disclosed	34.8
Caste	SC	11.0
	ST	12.9
	OBC	44.5
	Gen/Others	11.6
	Not disclosed	20.0
Total, N	**=**	**147**

### Data collection process

Detailed semi structured interview guides, tailored for each stakeholder category, were developed and translated into local languages - Kannada, Telugu, Assamese, and Hindi (Supplement file 1). The interviews were audio recorded with written/oral consent from participants after explaining the purpose, risks, and benefits of the study. Participants were provided with the option to ask for clarifications and to withdraw from the study at any time. Participation was voluntary and uncompensated. Privacy and confidentiality were maintained during interviews.

A two-step process of criterion sampling was used to shortlist the participants:

- Step 1: For each solution, separate line lists were created from program data for beneficiaries referred by community structures and who used the solution in the year prior to the survey. Based on program data, another list was created for other stakeholders (auto drivers, community leaders, NTEP staff, and ASHAs) in selected TUs and districts.
- Step 2: The project team contacted listed beneficiaries and stakeholders (up to three call attempts) to obtain verbal consent for participation. Calls continued until the desired sample size was reached. Rapport was established, and PwTB were asked to identify their primary caregivers.

Study tools were finalized after piloting them on field (2–3 interviews for each category). Final interviews were conducted with consenting participants at their residence or in a nearby health facility as per their convenience. The study explored background information, general TB history, BCS related knowledge/understanding, usage experiences, perceived benefits/challenges and recommendations for improvement. Facilitators and healthcare providers were separately interviewed to capture their perspective and experiences regarding feasibility of the solutions at the community level, scope of scalability, potential limitations and recommendations for future improvements. Each interview lasted for approximately 45–60 min.

### Quality control

Two field investigators (one male, one female, social science graduates and proficient in local languages) were recruited in each state to conduct interviews and translations. Qualified public health researchers supervised and monitored the investigators throughout the study period, with regular debriefing sessions, conducting random quality checks and providing continuous feedback. Investigators received two days of extensive training on qualitative data collection, ethics, tools, and study protocol. Investigators maintained field notes for transcription and analysis purposes. All audio files were provided individual id for anonymization and archived at the organization head office.

### Data analysis

A trained research team used a deductive approach and developed a pre-coded matrix using a shared coding framework. The initial codes were guided by the tools and the study objectives. Data from the IDIs were translated from local languages into English by trained translators directly into the pre-coded matrix. All translations were reviewed for accuracy and completeness by the central research team by cross-checking audio files. The final translations, together with field observation notes, were used for thematic analysis.

Phenomenological approach allowed the study to explore lived experiences around use of the BCSs among individuals from high risk vulnerable communities. Aligned with the study’s objectives to understand the use of solutions, it was observed that PwTBs and their caregivers adapted the interventions according to their individual conditions and life situations. The phenomenological approach provided insights into how these adaptations supported and sustained their treatment seeking journey in the context of TB.

The analysis started with a familiarization process with the data separately from users’ (PwTB, caregivers), facilitators’ (auto driver, CS leader) and healthcare providers’ (NTEP staff, ASHAs) perspectives. Findings from the users’ experiences, were further weighed alongside perceptions from other stakeholders. Initial codes were iteratively refined and organised into higher-order categories of themes by the research team. Codes reflecting similar concepts were grouped into categories, which were then abstracted into sub-themes and major themes. Thematic saturation was reached through a process of collaborative analysis, where the research team iteratively reviewed and discussed emerging patterns. This approach ensured that no new themes were being identified and that the findings thoroughly unpacked the experiences and perspectives relevant to the study's objectives represented in the pre-coded matrix. Triangulation across various types of participants was incorporated in analysis, where themes from different groups were compared and discussed to ensure consistency and depth in the findings.

### Reflexivity & positionality

The study was conducted by a multidisciplinary research team from KHPT with prior experience in tuberculosis and qualitative research. Their training and research experience helped to uncover the interaction between the innovative behaviour change solutions and the existing health services and further understand the possibility of how they could facilitate improved service uptake through user experiences.

National Institute of Mental Health and Neurosciences (NIMHANS, Bangalore’s Institutional Ethics Committee approved the study (No. - NIMHANS/40th IEC (BEH.SC.DIV.)/2023; March 20, 2023). Reporting of the study processes and findings adhere to Consolidated Criteria for Reporting Qualitative Research (COREQ) guidelines (Supplement file 2).

## Results

**Fig. 2:**
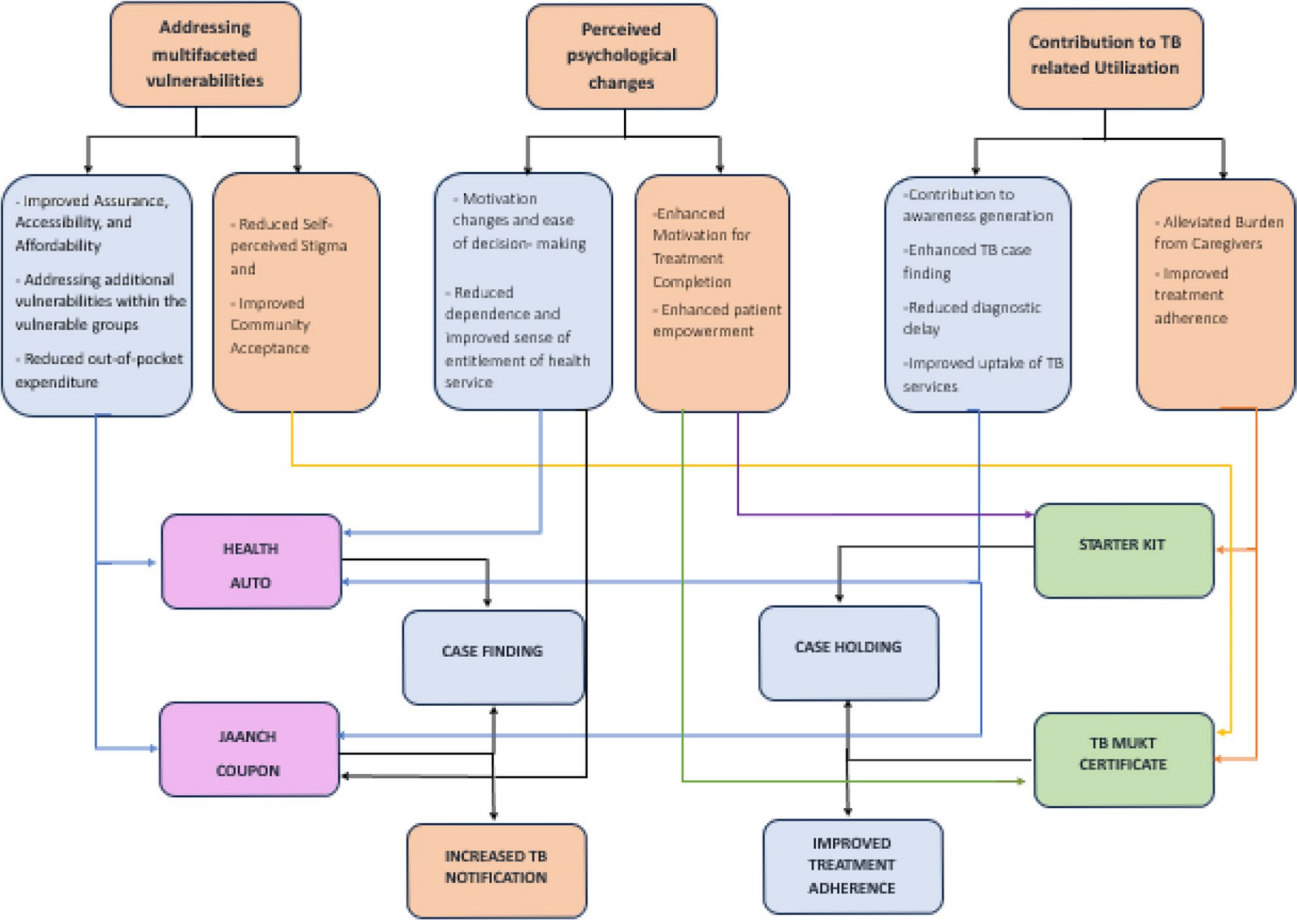
Conceptual representation of pathways influencing TB service uptake in the community

Guided by the analytic approach, the data were organized into major themes and sub-themes that reflect participants’ perspectives about the usefulness of behaviour change solutions and their potential influence on TB care-seeking among vulnerable groups. Based on the analysis, three major themes emerged which were reflective on the outcomes of the four BCS solutions viz. addressing multifaceted vulnerabilities, perceived psychological changes and contribution to TB related service utilization as depicted in Fig. 2. World Health Organization’s [17] availability, accessibility, acceptability and quality (AAAQ) provided a guiding framework for data interpretation. Given the demand side experiences as the focus of the study, its principles helped situate how cognitive and behavioural solutions addressed barriers and facilitated more equitable access to TB services. AAAQ was seen from the perspectives of the beneficiaries and their specific needs for TB care at a given time and situation. Since the BCSs did not create new TB related services, rather were designed to enhance community healthcare seeking, specific dimensions of AAAQ framework were not universally applicable.

### Health auto

#### Accessibility and affordability

Findings suggest that for many of the participants health auto improved ease of access to health facility. It ensured hassle-free, safe and free-of-cost travel which reduced their out of pocket expenditures for availing TB care services such as hospitals visits, medication and follow-up visits. Facilitators like auto drivers echoed similar views, further highlighting its convenience for a PwTB as compared to other public transportations.

*“I have used it around three times…once I went for check-up*,* second time for medicines and for report on the third time.” (Female*,* 19 years*,* user*,* Kamrup Assam)*.

*“More number of patients are using this and people waiting for this auto as they can’t spend Rs. 40–50. It is easier for patients to go for a test*,* to buy medicines and all”. (Male*,* 52 years*,* auto driver*,* Ballari*,* Karnataka)*

#### Addressing additional vulnerabilities within the vulnerable groups

Analysis revealed that health auto was more useful for people belonging to lower economic strata, people with limited mobility, older population in the community, housewives, young women and men who were out of work due to TB. As per all categories of stakeholders, absence of this solution would result in negligence of the symptoms, delay in diagnosis and disruption in treatment.

*“It was normal; it was for free. Whenever hospital staff asked to visit*,* I could go easily and come back home easily. Whenever I do not have money*,* I don’t have to think; when called*,* he will come”. (Female*,* 21 years*,* User*,* Hyderabad*,* Telangana)*

*Many TB patients are economically and physically weak…for them*,* these free auto services have been very useful in reaching the facility without inconvenience and extra cost.” (ASHA*,* Hyderabad*,* Telangana)*.

#### Supporting decision-making and motivation

The user accounts revealed that the auto drivers played a crucial role in motivating them to get tested. For instance, drivers’ polite and receptive behavior, treatment support, positive conversations, timely service, and handholding often encouraged presumptives to visit health facilities. This increased motivation, enabled PwTBs make quick decisions regarding their visits to health facilities, reducing decision fatigue.

*“If we are getting such free services*,* we feel good*,* as at this age it becomes difficult to go by ourselves. Before there were no such services and now*,* they have provided us for free. We need to thank them for this.” (Male*,* 60 years*,* User*,* Baksa*,* Assam)*.

#### Promoting autonomy and entitlement in care

The findings pointed that availability of on-demand auto service led to lesser dependence on others for accessing health facility and facilitated independent decision-making. Users shared that the drivers’ accompaniment provided a sense of entitlement and privilege at hospitals, compared to solo visits, as hospital staff prioritized their care.

*“It was good; the health staff prioritized me. When I went through this process*,* hospital staff prioritized me. It didn’t take much time for the check-up. It would have taken a lot of time if I went by myself for treatment. Because of this auto hospital staff took extra care of me”. (Male*,* 56 years*,* User*,* Mahabubabad*,* Telangana)*

#### Promoting TB awareness

According to the users, health auto facilitated TB awareness generation. They received relevant information related to the disease such as mode of transmission, testing, treatment, importance of treatment completion, nutrition etc., which helped them gain relevant knowledge.

*“The conversation with the driver was good. He would motivate me to go for a check-up without any fear. He would tell you - that it is just a cough; you don’t decide if it is TB; just go for testing*,* and even if it is TB*,* you have free service*,* so you don’t have to worry. Many take my auto for testing; after using medicines*,* they have recovered. You can also recover. He was reliable. He gave me a sense of safety. (Male*,* 56 years*,* User*,* Mahabubabad*,* Telangana)*

#### Service uptake and outreach

Healthcare providers pointed out that health auto resulted in considerable increase in footfall. Identifying the gap in service outreach in urban areas where number of ASHAs is less, NTEP identified the significance of the health auto. It was mentioned that this solution was able to address the needs of different vulnerable groups.

*“It is helpful in places where there aren’t a lot of ASHAs or places where the hospitals/health centres are far away. It’s also helpful for people living alone*,* and people living in slums who hesitate to come*,* for those people who go to duty at odd times*,* etc.” (Male*,* Healthcare provider*,* Bangalore*,* Karnataka)*.

#### TB case detection

Service providers like NTEP staff, ASHA and community structure leaders reported that health auto contributed to TB case finding. Given the fact that it helps in ferrying presumptive persons free of cost and on time, it helped in early diagnosis.

*“TB referral was happening before too*,* but the coordination with health auto has created some improvement. It has made it easier for some cases to be referred.” (Male*,* 52 years*,* NTEP staff*,* Bangalore*,* Karnataka)*.

Themes such as accessibility & affordability, service uptake & outreach and TB case detection reflect accessibility on how PwTBs navigated practical issues such as service reach, transportation and geographic barriers with the help of health auto. Availability was inbuilt in the solution as it provided beneficiaries with the awareness of existing TB services, as evident from the theme accessibility & affordability. Also, through a free-of-cost travel it reduced the direct & indirect costs of TB treatment and expanded their perceived options for TB care. Subsequently, themes such as addressing additional vulnerabilities within the vulnerable groups, supporting decision-making & motivation, promoting autonomy & entitlement in care and promoting TB awareness suggest that the solution was widely accepted by the beneficiaries. It shaped their willingness to avail TB services. Overall, the quality of the solution was ensured through its inclusive nature in terms of age, gender, economic status, health conditions and specific needs of the PwTBs. However, healthcare staff expressed concerns about long-term feasibility of the solution as budgetary constraints at the health system level may potentially limit the scope for scale-up and sustainability. Additionally, PwTBs and drivers raised concerns about stigma associated with being seen in a “TB auto” in spite of all its benefits.

### Jaanch coupon

#### Out of pocket expenditure

Data indicated that jaanch coupon helped in alleviating concerns about testing costs, especially for non-earning family members and those with lower incomes. One user noted that it helped her save money, as her husband’s wages were modest. Users of jaanch coupon also reported easy, and hassle-free access to service at the health facilities.

*“Initially*,* I had a fear of getting tested for TB. In private hospitals*,* testing was expensive and required a long waiting time. The jaanch coupon not only saved my money on testing but also significantly reduced the waiting time.” (Male*,* 30 years*,* User*,* Ballari*,* Karnataka)*.

#### Supporting decision-making and motivation

Data revealed that jaanch coupon was helpful to encourage presumptives to undergo TB testing, mainly because it saved additional costs and time at the health facility as compared to private facilities. Use of jaanch coupon also nudged the symptomatic persons to make informed choice regarding the place of test.

*“I was tired of receiving treatment at various places (hospitals) like Chanpatiya Betiah etc. Meanwhile*,* the doctor of Betiah told me that I have TB and prescribed medication. Upon returning home*,* when I attended a group meeting (samuh jeevika meeting)*,* I shared my situation with one chachi then she provided me with this coupon. Once I got this coupon*,* I visited the government hospital where I got free medicines*,* test and X-ray. Now I am feeling better”. (Female*,* 45 years*,* User*,* West Champaran*,* Bihar)*

#### Promoting entitlement in care

Users mentioned that jaanch coupon facilitated TB testing at the health facility on a priority basis, which in turn gave a sense of entitlement.

*“I found this to be very useful because the process of testing is quick and observing this*,* I believe the hospital management will try to provide quick services. They spared me from standing in the long queues”. (Female*,* 80 years*,* User*,* Mahabubabad*,* Telangana)*

#### Addressing diagnostic delay

Healthcare providers noted that jaanch coupon facilitated timely testing, especially for populations who are economically weak, and belong to hard-to-reach areas. It was also mentioned that people who previously preferred private facilities, now shifted to public facility. NTEP staff in Telangana noted increased symptomatic referrals and TB detection post use of jaanch coupons.

*“People coming for diagnosis has increased since the introduction of jaanch coupon”. (Male*,* 37 years, NTEP staff*,* Sangareddy*,* Telangana)*

#### Service acceptance and uptake

Participants stated that the jaanch coupon, due to its convenience, has shifted TB testing from private to public health facilities, especially in the high-risk areas. Community structure leaders noted increased demand and renewed trust on public health services among marginalized populations.

*“Previously*,* patients were more inclined to visit private hospitals*,* but now they are shifting towards public sector*,* thanks to the jaanch coupon. The testing rate has increased*,* primarily due to distribution of coupons”. (Female*,* 33 years*,* NTEP staff*,* Koppal*,* Karnataka)*

#### TB case detection

According to the service providers across intervention geographies, introduction of jaanch coupon resulted in an increase in the number of TB testing which in turn facilitated TB case finding.

*“With the coupon*,* notification has increased. Patients in hard-to-reach areas or far from facilities are now accessing diagnosis and treatment*,* whereas earlier many used to go to private providers or local unqualified practitioners.” (Female*,* 26 years*,* NTEP staff*,* West Champaran*,* Bihar)*.

Themes such as out of pocket expenditure, addressing diagnostic delay, service acceptance & uptake and TB case detection highlight that jaanch coupon addressed the barriers like service reach, and financial constraints increasing the accessibility to diagnostic services. Additionally, the solution empowered beneficiaries with the awareness of availability of public health services which is free of cost. Subsequently, themes such as supporting decision-making & motivation, promoting entitlement in care, and service acceptance & uptake reflect how the solution was universally available and widely accepted by the beneficiaries. It worked as a push factor for the symptomatic persons to go for TB test without hesitation. Overall, the quality of the solution was in its ability to be a catalyst to bring positive change in community mindset to seek timely diagnosis. However, concerns were raised by healthcare staff about some unintended effects of the solution. It was noted that indiscriminate distribution of coupons by community structures might potentially compromise the quality of referrals and could lead to additional burden on already limited resources. Some community leaders also observed that individuals refused to use jaanch coupon, largely due to persistent stigma around TB.

### TB starter kit

#### Enabling motivation for treatment adherence and completion

It was noted that the starter kit played a crucial role in preventing confusion and ensuring timely dosage. Further, it helped PwTBs in tracking side effects, and reducing the chances of missing medication. Both PwTBs and healthcare providers mentioned about the positive impact of the kit on treatment adherence. The visibility that the starter kit created, gave a sense of achievement amongst users.

*“With the starter kit*,* I never forget to take my medicines—the calendar keeps it in my mind every day*,* and the pictures make me feel good. It helped me track my progress and gave me a sense of reassurance about how far I have come.” (Female*,* 24 years*,* User*,* Dibrugarh, Assam)*.

#### Promoting patient empowerment for self-management

The healthcare providers stated that starter kit fostered self-management of treatment among PwTBs. They were able to take an active role in their treatment journey by monitoring symptoms, side-effects, day-to-day emotional status and in tracking progress. This provided PwTBs with increased sense of agency and led to better treatment adherence.

*“They get good understanding about TB*,* treatment*,* dietary requirements etc. since the introduction of starter kit. Many patients have told us that the starter kit has been very useful.” (Male*,* 38 years*,* NTEP staff*,* Baksa*,* Assam)*.

#### Addressing caregiver burden

Both caregivers and the health service providers noted that the starter kit alleviated the burden from caregivers by providing a structured approach to medication administration and symptom tracking. By reducing the need to remember minute details about treatment, helping in tracking the progress, the calendar prevented confusion about medication. This allowed caregivers to focus on other aspects of patient care and improve their overall wellbeing.

*“One may forget and accidentally take medicine twice and overdosage can lead to side effects. However*,* with the habit of looking at the calendar*,* such instances of overdosage can be prevented without anyone reminding about it’. (Male*,* 23 years*,* Caregiver*,* Sangareddy*,* Telangana)*

The themes such as enabling motivation for treatment adherence & completion, promoting patient empowerment for self-management and addressing caregiver burden mostly addressed the acceptability and quality of services. The starter kit provided PwTBs with a visual aid which made treatment completion more tangible and personalized experience through easy tracking. Further it reduced dependence on caregivers which promoted acceptability. While the starter kit supported treatment adherence, those with limited literacy and understanding of the kit, found it less user-friendly pointing to quality issues.

### TB mukt certificate

#### Enabling motivation for treatment adherence and completion

Analysis revealed that the certificate served as a powerful inspiration that encouraged PwTBs to follow protocols and complete treatment. Both PwTBs and healthcare providers noted that receiving TB-mukt certificate raised awareness on curability of the disease, fostered motivation to complete treatment, and created a sense of achievement.

*“Those patients who were present there at the time of issuing TB mukt certificate*,* expressed positivity about my recovery. I told them that they too would recover soon and inquired about their medication adherence. My successful treatment brought them a sense of satisfaction” (Male*,* 54 years*,* User*,* Kamrup*,* Assam).*

*Upon receiving the certificate*,* patients began telling those around them that by adhering to their medication regimen*,* others too can achieve recovery and earn the certificate.” (Male*,* 40 years*,* NTEP staff*,* West Champaran*,* Bihar)*.

#### Addressing stigma and promoting social integration

Users pointed out that the certificate helped to dispel misconceptions about the disease and reduce stigma associated with TB. It had two-fold advantage in the context of addressing stigma - at the personal and at the community levels. Certificate by competent authorities helped provided a sense of validation and acceptance for PwTBs, allowing them to reintegrate into community without fear of discrimination. It helped PwTBs re-join school/college/work, travel, and get back to social life.

*“Earlier*,* even those from my own community (biradari) avoided my house fearing I had TB*,* and I also stayed away. But after completing treatment and receiving this certificate*,* they visit me without hesitation. Educated people understand once they see the certificate*,* and I show it to anyone who still doubts.” (Female*,* 45 years*,* User*,* West Champaran*,* Bihar)*.

Acceptability and quality components were noted from the themes - enabling motivation for treatment adherence & completion, and addressing stigma & promoting social integration. The certificate mainly helped PwTBs with a tangible goal to complete TB treatment, deal with community stigma and social assimilation post treatment completion highlighting its acceptability. Although the TB-mukt certificate was valuable, there was a perception that its utility to address stigma is more individualistic than community specific. It was emphasized that the certificate alone was often insufficient to motivate treatment adherence; rather, repeated counselling by health workers in combination with the certificate, was more effective in influencing behaviour change among PwTBs.

## Discussion

**Fig. 3:**
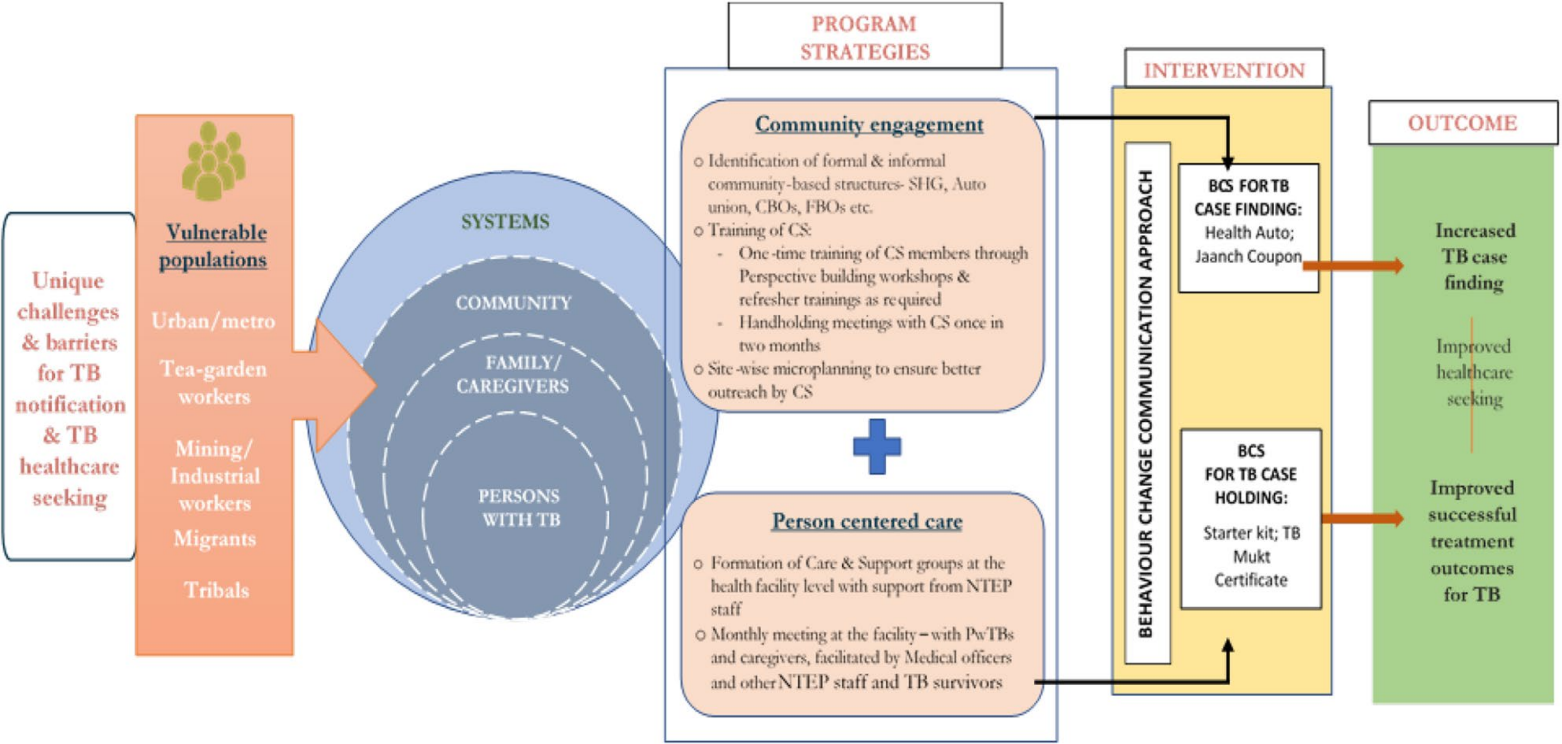
Theory of change explaining the implementation of BCS through community engagement and person centric care approaches to increase TB notification and improve treatment adherence

The salient findings from the study underscored the feasibility of four innovative behaviour change solutions - health auto, jaanch coupon, TB starter kit, and TB mukt certificate in four Indian states. The results highlighted that “nudging” cognitive biases among PwTBs and high-risk populations can influence health-seeking behaviour at the larger community level. Implemented through community structures and person centric strategies (CSG at the PHIs), BCSs were able to address specific barriers to healthcare seeking experienced by vulnerable populations and lead to enhanced TB case finding and treatment adherence, as depicted in Fig. 3.

The health auto addressed practical barriers to TB care like affordability, accessibility and decision fatigue. It demonstrated potential to improve service uptake and TB case finding, especially among rural and slum populations, which in turn positively influenced TB health-seeking behaviour. To elaborate, as per the theory of planned behaviour [18], health auto likely increased PwTB’s perceived control over care seeking. On the other hand, per social cognitive theory (SCT) [19], observing others use health auto might have motivated presumptive people in quick decision making. However, healthcare staff expressed concerns about sustainability and scalability due to associated cost and resource implications. The local adaptation of health auto as a “TB auto” indicates that inherent TB stigma can hinder optimal use of the solution.

Meanwhile, jaanch coupon addressed financial barriers and diagnostic delay. It also improved the motivation for TB testing, facilitated easy decision-making, and improved perceived entitlement to health services. Service providers noted reduced diagnostic delays, improved service uptake and enhanced TB case finding. As per the theory of reasoned action [20], the coupon likely improved attitudes toward care seeking by providing an idea of tangible benefits, reducing stigma and fear. Using a gain-framing approach [21], it may have shifted community’s perceptions. Per SCT [19], the coupon might have acted as a social cue to normalize TB testing. However, feedback from healthcare staff suggested a need to ensure referral quality to avoid overloading testing facilities leading to higher turn-around-time.

The TB starter kit was able to structure TB treatment journey by improving adherence, reducing default rates, and facilitating treatment continuation. It also reduced caregiver burden by promoting self-management through treatment tracking. Per the concept of cognitive behavioural therapy (CBT) [22], the starter-kit likely helped PwTBs develop adaptive coping strategies and reduce anxiety, fostering self-efficacy and empowerment leading to positive change in behaviour. While acknowledged positively, caveats included over-reliance on facilitators and lack of user-friendliness for illiterate populations.

The TB mukt certificate aided PwTB’s return to routine life post-treatment, motivating treatment completion, improving community acceptance, and reducing self-stigma. It likely served as positive reinforcement. Per observational learning [19], exposure to positive role models i.e. TB survivors may facilitate treatment adherence. Recognition for treatment completion might have boosted social status, sustaining positive behaviour as explained in SCT [19]. However, healthcare staff noted that stigma reduction is a gradual process that requires higher exposure time. Service providers suggested that effectiveness of the certificate cannot be seen as a stand-alone factor as treatment adherence and completion also depends on rigorous support for enhanced motivation.

Integration of the emergent themes to the AAAQ framework demonstrates that the lived experiences of primary stakeholders captured through the four behaviour change solutions reflect interconnected factors of TB care continuum. The synthesis reveals how beneficiaries and service providers interpreted, valued, and sometimes challenged these solutions within the realities of their everyday lives. For example, the health auto and jaanch coupon increased service uptake as intended by programme design, expanded perceived *availability* of treatment options and reduced physical and financial barriers that previously limited their *accessibility* to TB testing and treatment. Likewise, the starter kit and TB mukt certificate highlighted that motivation, autonomy, and social integration mattered as much as material support. Importantly, this process enabled to uncover specific but crucial shortcomings. Persistent TB stigma at the community level reduced the *acceptability* of the health auto and jaanch coupon. Literacy barriers undermined the usability of the starter kit, and the TB mukt certificate’s utility to address stigma remained constrained to an individual level than normative change at a broader community level. Further, concerns from healthcare providers about long-term feasibility, and standard of referrals highlighted system-level challenges that could potentially affect the *quality* of the solutions.

### Limitations of the study

There are few caveats that need to be acknowledged. Firstly, findings of the study cannot be generalized because it was a qualitative study conducted with vulnerable population groups to explore specific user experiences and service providers’ perspectives on the solutions. Secondly, social desirability bias could be a potential limitation which might have influenced the users’ and service providers’ responses. Lastly, the study has to be seen in the purview of the intervention/program as it attempted to understand the barriers and facilitators for the utilization of the solutions and had limited scope to explore deeper interlinkages to the causes of acceptance of any solution.

However, this study adds to the scarce literature on BCS in TB care seeking among vulnerable populations. While social and behaviour change (SBC) strategies have been widely used in other health fields [23–25], this is a rare attempt to address the evidence gap for TB in India. These solutions have potential for scale-up via NTEP, contributing significantly to person-centered care approach of national TB program. Going forward, it is important to recognize that the effectiveness of BCSs depends on the specific population and associated contextual factors such as cultural nuances, language differences, differential awareness, and infrastructural limitations which can significantly influence outcomes. A gap analysis is therefore crucial to identify where BCSs are most needed, determining if solutions can function standalone or require integration. Contextualization and tailoring of intervention timing and frequency are essential. Policymakers should consider integrating these strategies into national programs, allocating resources, and building community partnerships.

## Conclusions

There are no proven models or approaches that have demonstrated success in preventing TB within the unique socio-cultural contexts of vulnerable populations. This study presents compelling evidence for BCS implementability in improving TB care-seeking and adherence among vulnerable populations in India. Overall findings suggest that successful implementation plan need to consider the unique socio-cultural contexts of the vulnerable groups and base solutions on specific health needs. The study further highlighted that beyond existing IEC and advocacy, communication & social mobilization (ACSM) strategies, the BCS interventions opens a range of opportunities to address a multitude of issues and barriers related to TB care seeking, which otherwise had remained unexplored so far.

## Supplementary Information

Supplementary Material 1.

Supplementary Material 2.

## Data Availability

All relevant data are included within the manuscript or supplementary information. Audio files and transcripts of this study contain sensitive and personal information about persons and families and thus will not be shared to maintain participant confidentiality.

## Abbreviations

TB: Pulmonary tuberculosis
NTEP: National TB Elimination Program
NSP: National Strategic Plan
BTB: Breaking the Barriers
BCS: Behaviour Change Solutions
IDI: In–depth Interviews
BCC: Behaviour Change Communications
TU: Tuberculosis Units
CS: Community Structures
CSG: Care and Support Groups
SHG: Self Help Group
CBO: Community Based Organization
FBO: Faith Based Organization
ASHA: Accredited Social Health Activists
SCT: Social Cognitive Theory
CBT: Cognitive Behavioral Therapy
PHI: Primary Health Institutions
IEC: Information, Education and Communication
PwTB: Persons with TB
AAAQ: Availability, Accessibility, Acceptability and Quality framework
SBC: Social and Behaviour Change
ACSM: Advocacy, Communication & Social Mobilization
