# Supplementary material for: Behaviour change solutions driven by cognitive insights for improving TB health care seeking among vulnerable population: an exploratory multi-state qualitative study in India
Source: BMC Public Health. 2026 Feb 12;26:921. doi: 10.1186/s12889-026-26542-x (PMC12997947; doi:10.1186/s12889-026-26542-x)
Supplement: Supplementary file 1 — Supplementary Material 1. [file 12889_2026_26542_MOESM1_ESM.pdf]

IN-DEPTH INTERVIEW GUIDE

Understanding user-feasibility of behaviour change solutions for persons with Tuberculosis in community engagement program in the states of Karnataka, Telangana, Bihar & Assam under the BTB Project

NAME OF STATE: ___________ NAME OF DISTRICT: ___________ NAME OF TU: ___________

IDI SERIAL NO. ____________

SELECTED VULNERABLE GROUP (RECORD RELEVANT CODE): _____________ [1- Urban group, 2- Migrant group, 3-Mining group, 4-Tea garden worker, 5-Tribal group, 6- Industrial group, 7-Urban metro (only for Bangalore/Hyderabad)]

DATE OF THE INTERVIEW (DD-MM-YYYY): _______________

INSTRUCTION:

Please read the subject information sheet and obtain informed written/verbal consent from each of the participants before starting the in-depth interview. Please ensure that except for the interviewer(s) and the respondents, no other people are present during the interview.

<u>Background information sheet</u>

1. BCS solution used:
2. Name of the participant:
3. Stakeholder type: PwTB/Caregiver/CS leader/Auto driver/TB buddy/TBC/CS leader/NTEP staff (DTO, MO, STS, STLS, LT)/FLW (ASHA/ANM)
4. Nikshay ID (ONLY for PwTB / Caregiver of PwTB):
5. Sex: Male / Female / Transgender
6. Age (in completed years):
7. Place of residence: Urban / Rural
8. Date of diagnosis (ONLY for PwTB):
9. Date of start of the current treatment (ONLY for PwTB):
10. Type of TB (ONLY for PwTB): DS-TB / DR-TB
11. Site of TB (ONLY for PwTB): Pulmonary / Extra-pulmonary
12. Place of treatment (health facility name) [ONLY for PwTB]:
13. Level of education: Illiterate/below-primary/secondary/higher secondary/Graduate and above
14. Religion: Hindu/Muslim/Christian/Others/Not mentioned
15. Caste: SC/ST/OBC/GEN/Not mentioned
16. Use Tobacco [only for PwTB]: Yes/No
17. Consume Alcohol [only for PwTB]: Yes/No

Study guide

Thank you for participating in this interview and we would like to learn from you about the usefulness of behaviour change solutions for the population susceptible to TB - (Please read one of them according to the specific vulnerable group targeted in the district: mining workers/industrial population/tea garden workers/ tribal/ migrants/ urban group and urban metro city population).

BCS 1: HEALTH AUTO (HA)

Objective: Addresses the hidden costs, making health care accessible & addressing decision fatigue

BENEFICIARIES/USERS: [WHOEVER HAS USED THE SERVICE IRRESPECTIVE OF +VE OR -VE]

Note: *Display Health Auto guide/picture first and start conversation*

1. Have you ever heard about any such auto service in your locality which takes people to health facilities for TB treatment? If they say yes, probe further [Check: As the respondents are already beneficiaries, no one is supposed to say 'NO' or keep silent – if that is the case, please explain more about the solution to help them understand what the interviewer is talking about and try to get responses]
2. How and when did you come to know about health auto? (*Probe*: Family/relatives/friends/neighbour/community coordinator/CS leader/any other)
3. What all information did you receive about Health Auto?
    - Can you tell us, how many times have you used the auto service during your treatment journey?
4. For what purposes have you used the auto? – *(Probe: traveling to hospital for test/fetching medicines/attending CSG meeting/any other, please specify…..how many times PwTB used this services)*
5. Can you tell us, how was your experience of using the health auto service? Please explain….
6. Do you think using health-auto was beneficial? If yes, in what way did the health-auto benefit you?

   – *(Probe if respondent is not able to comprehend: Easy to reach facility/time saving/saved money/any other benefits, please elaborate)*
    - How was your experience of communication with the auto-drivers during the trips? [*Probe: Awareness/information received on TB symptoms, test & treatment from the auto drivers; details of information received*]
    - After using Health auto service, how did this service affect your perception around TB? (*Probe: was the respondent concerned about status disclosure to people around, what they will think or say about them, did anybody ask questions about the auto service you are availing*) [in addition to the previous question, this will dig further into self-stigma]
7. Did you face any challenge/difficulty/problems while using health auto? Would you please elaborate to us the various challenges you faced? – *[Probe: concerns on safety, privacy/concerns about status disclosure/auto drivers behaviour (friendly or rude) / availability of auto when needed or called / waiting time / Any other…. for any of answers please encourage respondent to elaborate the reason why they felt so]*
8. Can you tell us, what did your family/peer/neighbours/colleagues have to say about health auto service? How did they look at the facility? Please elaborate.
9. Would you recommend the service to family/peer/neighbours/colleagues or anyone with TB symptoms? If yes, why do you feel that other people also should use the service? (Open ended)

10. Would you like to give any suggestions - in what ways the auto service could be made more person friendly? *[Probe: readiness of auto drivers; gender preferences; drivers to be more sensitized; popularize the service in the community; behaviour/attitude of driver; IEC design; any other mode of transport that can be used for this service; any other suggestions]*

SERVICE PROVIDERS (NTEP/FLW)

1. Are you aware about the Health auto service being implemented in your area? If yes, can you please tell us for what purpose it is used (what all services it is providing)? *(encourage to speak in detail)*
2. Do you think HA is useful in any way, for TB related services in your community?
   - If yes, can you please elaborate what are the specific usefulness of this auto service? *[Probe: Usefulness for referrals, testing, notification from vulnerable population / reaching the difficult to reach groups/Usefulness in reducing patient delay in terms of getting tested, treatment initiation/ transportation of sputum samples wherever necessary / benefits for PwTB in terms of motivation/adherence/de-stigmatization etc./Any other, please elaborate]*
3. What are the challenges or drawbacks in your opinion? Please elaborate…(Open ended)
4. In your opinion, is there a scope for improvement and scale-up as part of NTEP? (Open ended)

SOLUTION PROVIDERS (AUTO DRIVERS)

1. How long have you been engaged with this service? -in days/months/years. Please tell us how did you get involved with this service?
2. Think about the time you were first approached by the program team and tell us how prepared or open were you to get engaged in the services?

   What motivated or triggered you to be engaged in this service? Please elaborate. - (*Probe: Monetary benefits/ community service/both/any other*)
3. Were you given any orientation about TB by the program team? What all did you learn from them about TB…please explain. - *[Probe: what is TB, symptoms, tests, testing facilities, treatment and duration of treatment, care & support groups etc.]*

   Were you oriented about your roles and responsibilities by the program team? What information did you get form them…please elaborate?
4. On an average, how many trips do you have to take in a week? – [in numbers]
5. Can you please tell us about how you feel to serve as a health auto driver? (probe: confidence / self-worth / accomplishment to be able to serve community / satisfaction when people get cured / value addition to the society / feeling wanted or important in the community / any negative feeling like – personal emotional turmoil to see PwTB/Caregivers' sufferings / any other…)
6. What were the barriers or challenges, if you had faced any? – [Probe: support/encouragement from family, neighborhood, friends etc.]
7. Did you face any challenge while in service of a health auto? If yes, can you please elaborate for us what challenges have you faced? - *[Probe: Distance/monetary issues or timely receipt of honorarium, time management, high demand limited no. of autos, those availing the service are reluctant to use, rude behaviour of PwTB/caregivers,* any stigma with the fact that the auto is used for TB related services, general population hesitant to use your auto for general purposes / any other…please elaborate*]*
8. In your opinion, how accepting/welcoming were the health staff towards you or the patients you transported? - Persons ferried through the health auto were attended to immediately/health staff appreciative towards the driver/health staff did not pay much attention/health staff refused to attend the patients/any other, please specify

9. Would you like to give any suggestion(s) to improve the service? - [*Probe: increase no. of autos to align with demand in an area, fix an honorarium; other acknowledgements you might get]*

CS LEADER

1. How do you think HA has been useful in the work that you do?
2. In your opinion what is the general perception about HA in the community? – (Probe: are open or e hesitant/apprehensive of use of HA, and also those using the HA)
3. How is the acceptance for HA in the community? Can you help us understand? - (Encourage participants to share stories about how it has helped)
4. How many presumptives have you come across so far? How many referrals have you made so far through HA? What happened after that? - *[Probe: did they use it for test?]*
    - Has anyone availed auto service for attending CSG meetings, how often do they use?
    - Has people used HA for follow-up and obtaining medicines?
    - Please share your experiences in details.
5. Do you see any increase in demand for HA in the community? Can you elaborate. - *[Probe for – more number of people coming and enquiring about HA and TB testing]*
6. In your opinion how is the perception of people about HA in health facility? – [*Probe: People hesitant/apprehensive of use HA, attitude of health staff towards auto-driver and CS leaders, and also the PwTB availing HA]*
7. How is the acceptance for HA in the health facility? Can you help us understand? - (Encourage participants to share stories about how it has helped)
8. Going further, would you like to give any suggestions to us to improve HA? Can you please elaborate?

BCS 2: JAANCH COUPON (JC)

Objective: To be used by frontline workers and supervisors at work in creating an easy referral program, reduce delays in diagnosis, increase case finding

TB PRESUMPTIVES (WHOEVER HAS USED THE SERVICE IRRESPECTIVE OF +VE OR -VE)

Note: *Display the solution to the respondent(s) to begin the conversation*

1. Have you seen this (after displaying)? Can you tell us what this is called?
   - If yes, from where did you come to know about JC? When did you come to know about
   - what it is used for, can you explain about this?
2. Have you ever used Jaunch coupon? If yes, in your opinion what is the use of Jaunch coupon? *(Probe: Got motivation to go for test/testing costs/whether it is helping at the hospitals/any other)*
3. How did it help you? Can you please elaborate to us*? - (Probe: ease of accessing diagnosis facility, testing services)*
4. Within how many days of receiving the JC, did you go for testing? If not used immediately, probe for reasons for delay.
5. Did you face any challenge while using the JC? If yes, can you please elaborate what challenges you faced? – *(Probe: Hesitation in carrying the coupon/usage not properly explained by CS/FLW/CC/not seen as reliable by family/peers/not accepted at the hospital/any other)*
6. What does your family/peer/neighbours/colleagues have to say about the JC? Please elaborate.
7. Would You recommend anyone with TB symptoms to use it? If yes, please explain why would you recommend? (Open ended)
8. Going further, would you like to give any suggestions to us to improve JC? Can you please elaborate?

SERVICE PROVIDERS (NTEP/FLW)

1. Are you aware of the Jaunch Coupon service being implemented by KHPT? Can you please explain?
2. In your opinion, to whom it is to be given? *[Probe: If they respond it is to be given on a priority basis, explore why do they think so]*
3. What difference has it made, according to you (If any)?
   - What difference to the PwTB's attitude
   - Differences in the attitude of caregivers/family members
   - Differences in the health workers' attitude
4. Can you please elaborate on what all benefits does JC have? – *[Probe: any change in referrals, testing and diagnosis, from vulnerable community/any effect on the patient delay/ people are availing public health facility more than private/ any other. If there is a change, why it is so?]*
5. What is the opinion of the doctors/other staff in the facility on JC? Are they open to using it?
6. Do you feel that this service has a scope to be adopted in NTEP and scaled up as part of the existing referral system? Please elaborate - why you feel so?
7. Going further, would you like to give any suggestions to us to improve JC? Can you please elaborate?

SOLUTION PROVIDER (CS LEADERS)

1. How do you think JC has been useful in the work that you do?
2. In your opinion what is the general perception about JC in the community? – [*Probe: are open or hesitant/apprehensive about JC, and also those using the JC]*
3. How is the acceptance for JC in the community? Can you help us understand? - (Encourage participants to share stories about how it has helped)

4. How many presumptives have you come across so far? How many referrals have you made so far through JCs? What happened after that? *[Probe: did they use it for test?]*

5. Do you see any increase in demand for JC in the community? Can you elaborate? - [*Probe for – a greater number of people coming and enquiring about JC and TB testing]*

6. In your opinion how is the general perception about JC in health facility? – *[Probe: People hesitant/apprehensive of use of JC, attitude of health staff towards those who are distributing the JC, and also those using the JC]*

7. How is the acceptance for JC in the health facility? Can you help us understand? (Encourage participants to share stories about how it has helped)

8. Going further, would you like to give any suggestions to us to improve JC? Can you please elaborate?

BCS 3: TB MUKT CERTIFICATE (TB FREE CERTIFICATE)

Objective: End the TB journey on a positive note, gear up for life ahead, & fight stigma

PERSONS WITH TB/CAREGIVERS (USERS)

Have you ever attended any care & support group (CSG) meetings conducted at the health facility? Can you recall any meeting you attended and tell us in detail –

- what was discussed?
- how did you feel attending the meetings?
- Was attending such meetings helpful during your treatment journey? *[Probe: encourage the respondent to share stories/experiences in detail]*

Note: *Display a copy of TB Mukt certificate to the respondent(s) to initiate the further conversation*

1. In such meetings, have you ever heard of such a certificate being given to PwTB, who have successfully completed their TB treatment? If yes, can you please tell us what you know about such certificates.
2. Have you seen anyone, earlier during your treatment journey, receiving a TB mukt certificate? *(Encourage the respondent give details)*
3. Do you know how this certificate helps? How did it helped the others whom you know, who received the certificate? – [*Probe: Express the experience of others, as they might have witnessed]*
4. Knowing about this TB mukt certificate, were you anyway motivated during your journey? If yes, what was the motivation all about? Can you please tell us more about it? – *[Probe: how it motivated you?]*
5. Have you completed your treatment? When did you complete?
6. Have you ever received any such certificate? If yes, when did you receive the certificate? Can you please explain? – *[Probe: Where did you receive, who gave it, describe how it was given, how did others respond when you received the certificate, what did you do with the certificate]*

   [Check: As the respondents are already beneficiaries, no one is supposed to say 'NO' or keep silent – if that is the case, please explain more about the solution to help them understand what we are talking about and try to get responses]

7. How does having a TB Mukt Certificate make you feel? [*Probe: encourage the participant to express more how did they feel, how it helped them (if at they think it helped), relieved to have completed the treatment, free of a sense of fear, celebratory, change in perspective towards - TB/TB-treatment completion/contributing as a champion to the community, do you feel wanted and being treated better by others etc.*
8. Do you feel this certificate was helpful in any way? If yes, can you please tell us in which way did the certificate help you? – (Encourage stories)

   Probing points:

   - *Lesser discrimination by family/friends/community members,*
   - *Increased confidence and self-esteem to lead a normal life again*
   - *Helped in joining back job/school/college*
   - *Helped in finding suitable match for marriage (for unmarried girls)*
   - *TBM certificate is seen as a valid proof of treatment completion by the community/employers/peers/neighbours/ school -college authorities)*

9. Would you like to suggest anything for further improvement? Can you please elaborate? - *[Probe: can virtual certificate be more helpful/easy to carry, anything else?]*

SERVICE PROVIDERS (NTEP/FLW)

1. Are you aware of the TBM certificate being given to PwTB who have completed treatment? Can you please explain? - [*Probe: what it is, where it is given, why is it given]*
2. Have you ever attended any meeting where TBMC is provided? What was your experience of participating in these meetings (please explain)
3. Can you please elaborate on the usefulness of TBM certificate for the TB survivors? (Open ended) - *[Probe: any changes among the recipients in terms of confidence/attitude/stigma-discrimination towards TB, does it help them to rejoin work, resume classes, marriage, improve overall acceptance, etc.]*
4. Do you see any change in people's attitude towards treatment adherence and completion, knowing that they will receive a TBM certificate at the end of treatment completion? (Open ended) - *[Probe: elaborate on what kind of changes]*
5. In your opinion, what and how does TBM certificate contribute to the larger NTEP goal of TB elimination? (Open ended)
6. Do you feel there is a scope of integrating the TBM certificate in the existing treatment protocol? If yes, please elaborate (Open ended)
7. Would you like to suggest anything for further improvement? Can you please elaborate? - [*Probe: can virtual certificate be more helpful/easy to carry, or anything else?]*

BCS 4: TB STARTER KIT (SK)

Objective: Make people feel in control of their own treatment journey, expectation setting at the start of the treatment

PERSONS WITH TB (USERS) (PREFERABLY SELECT PWTB FORM THOSE WHO HAVE COMPLETED AT LEAST 3 MONTHS OF TREATMENT AND/OR SOMEONE WHO COMPLETED TREATMENT AND METICULOUSLY USED SK)

Note: *Display the starter-kit to the respondent(s) to begin the conversation*

1. *Have you seen this starter kit? [Display to the respondent as an opening point of the discussion] what do you call this? Where did you get the information about starter kit from?*
2. Have you received it? Did anyone explain to you how to use the kit? Did you find it relevant?
    a. If yes, what according to you are the usefulness of a starter kit? – *[Probe: helps keeping a track of medicine doses/motivates to continue treatment/makes me feel that treatment completion is achievable/home remedies help in treatment management/can easily record how I feel on a day to day basis/ managing emotions on a day to day basis is easier/any other]*
3. How do you feel this kit is useful in your day to day treatment adherence? – *[Probe: ADR, self-care, avoid missing doses etc.]*
4. Does this starter kit motivate you? Do you feel encouraged to see how you are progressing on your journey?
5. What do others (family/friends/colleagues/neighbours) feel about the starter kit? (Open ended)
6. How comfortable are you using the starter kit? Did you face any challenges? Please elaborate - [*Probe: User friendly or not, received training/orientation to use it]*
7. Is there anyone who comes and talks to you regarding the use of SK and supports you? When was the last time did someone come to talk to you about this? Can you share about that? Would you recommend anyone else in family/friends/neighbours with TB to use the kit?
    a. If yes, can you elaborate the reasons?
8. Going further, would you like to give any suggestions to us to improve SK? Can you please elaborate?

CAREGIVER

1 Are you aware about the Starter kit (TB calendar)? Please tell us what you know about it? – *[Probe: what it is, what it is used for, why is it used etc.]*

2 Where did you learn about SK from? Please explain, if you have received any orientation on how to use it? Can you please elaborate?

3 Please tell us, how the starter kit (TB Calendar) is helping you? – *[Probe: in supporting the PwTB, remember medicine doses, track PwTB's feelings and support them accordingly etc.]*

4 Thinking about a situation where there was no Starter Kit – do you feel, having this calendar makes a difference? If yes, would you please tell us what kind of difference do you feel it has made? – [*Probe: Are you better able to manage the PwTB? Is it helpful?]*

SERVICE PROVIDER (NTEP/FLW)

1 Have you ever come across the starter kit being implemented by KHPT? Yes/No

2 If yes, who introduced you to the kit? (KHPT staff/other health staff/any other

3 Were you given a demonstration of how to use the kit? Yes/No

4 In your opinion, how useful is this kit for a patient? - reduce LFU/easy to remember doses, easy to track missed doses/easy to follow-up with patients/better management of emotions during treatment/any other

5. By using this kit, do you see any change in health seeking behaviour of persons with TB? Yes/No
6. If yes, please elaborate (Probe: people not missing doses, completing treatment, etc.)
7. Do you feel starter kit is beneficial for NTEP in any way? Yes/No
8. If yes, can you specify how it may help in achieving NTEP’s goal to eliminate TB? (Open ended)
9. Would you like to give any suggestion for further improvement of the solution?

THANK YOU
